# Small intestinal adenocarcinoma accompanied by lynch syndrome: A case report

**DOI:** 10.1097/MD.0000000000035323

**Published:** 2023-09-29

**Authors:** Kyoung Won Yoon, Jaemin Jo, Donghyoun Lee

**Affiliations:** a Division of Critical Care, Department of Surgery, Chung-Ang University Gwangmyeong Hospital, Chung-Ang University College of Medicine, Gwangmyeong, Republic of Korea; b Division of Hemato-Oncology, Department of Internal Medicine, Jeju National University Hospital, Jeju National University College of Medicine, Jeju, Republic of Korea; c Department of Surgery, Jeju National University Hospital, Jeju National University College of Medicine, Republic of Korea.

**Keywords:** case report, lynch syndrome, microsatellite instability, small intestinal cancer

## Abstract

**Rationale:**

Lynch syndrome is caused by germline mutations of DNA mismatch repair genes. A significant risk increase for several types of cancer is one of the characteristics of lynch syndrome.

**Patient concerns:**

A 45-year-old female presented to the emergency department with abdominal pain that had persisted for a month.

**Diagnoses:**

The abdominal and pelvic computed tomography scan showed edematous and thickening of the proximal small bowel wall, as well as dilatation of the proximal bowel and stomach.

**Interventions:**

Tumor resection of the small bowel was performed, and adenocarcinoma was confirmed pathologically. Microsatellite instability was also confirmed.

**Outcomes:**

Postoperative imaging revealed soft tissue lesions with potential for tumor seeding. Two months after the first surgery, a secondary surgery was performed as a result of cancer recurrence. The patient received chemotherapy with capecitabine. The latest computed tomography scan, performed 19 months after the cessation of chemotherapy, did not show any recurrence.

**Lessons:**

In the rare incidence of small bowel cancer genetic mutation testing and detailed family history should be actively considered.

## 1. Introduction

Lynch syndrome (LS) is an autosomal-dominant disorder resulting from abnormalities in DNA mismatch repair genes and is associated with a heightened susceptibility to various types of malignancies.^[1,2]^ In LS, approximately 90% of mutations are found in the MLH1 or MSH2 genes, whereas approximately 10% are attributed to the MSH6 and PMS2 genes. Patients with LS have an elevated lifetime risk of developing various malignancies, notably including colorectal and endometrial cancer. However, they are also at an increased risk for cancers affecting the other gastrointestinal tract, ovaries, and urothelium.^[3]^ LS is diagnosed established by identifying familial clustering patterns and mutations in the DNA mismatch repair (MMR) genes. While LS has traditionally been recognized as primarily associated with colorectal cancer (CRC), it is noteworthy that approximately one-third of LS patients also develop extra-colonic malignancies.^[4]^ Small bowel cancer (SBC) is the predominant extra-colonic malignancy in over 30% of individuals with LS and tends to develop at a younger age.^[5,6]^ A significant proportion of advanced-stage SBCs are attributed to insidious presenting symptoms, leading to delayed diagnosis.^[7]^ Here, we present a case of LS that primarily manifested in the small bowel, emphasizing the importance of tailored surveillance strategies based on specific gene mutations.

## 2. Case presentation

**Figure 1.**
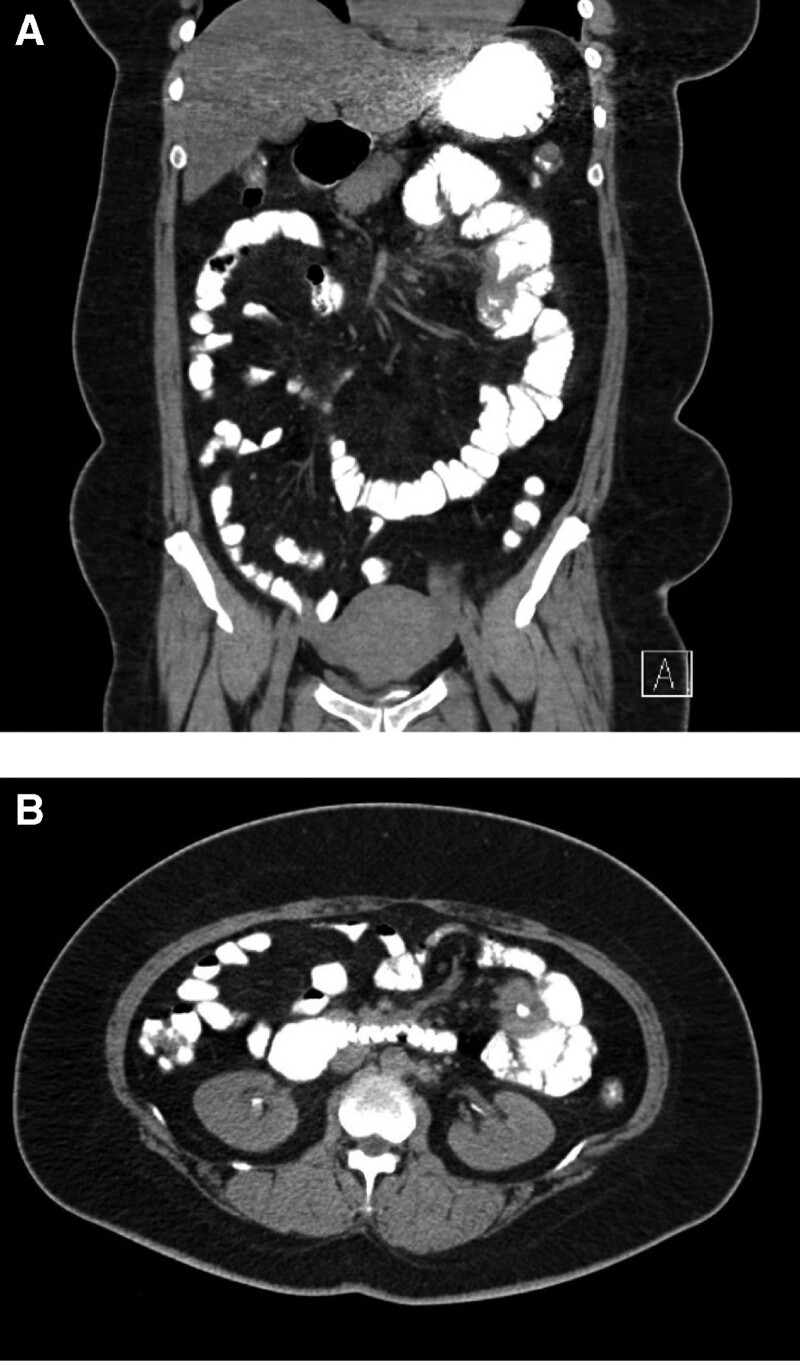
Pre-operative abdominal CT scan. CT = computed tomography.

A 45-year-old woman presented to the emergency department with abdominal pain. She complained of severe cramping abdominal pain that had persisted for 1 month, accompanied by intermittent vomiting. Two days prior to presentation, the pain increased in intensity. The patient’s medical history included an open appendectomy in 2009 and a myomectomy in 2015. The patient’s grandmother and 2 aunts had a history of colon cancer. Physical examination revealed severe tenderness and upper abdominal distension. Her vital signs included a temperature of 38.1ºC, heart rate of 98 beats/min, respiratory rate of 24 breaths/min and spot oxygen saturation of 98% in room air. Routine laboratory tests showed mild leukocytosis (12.1 × 10^9^/L) with an elevated C-reactive protein (13.2 mg/L). Serum tumor markers, including carcinoembryonic antigen and carbohydrate antigen 19–9, were within normal limits. No abnormalities were found in the liver function and urine analyses. Abdominopelvic computed tomography (CT) scan revealed a proximal jejunal tumor causing intestinal obstruction with an inflamed mesentery, including multiple enlarged nodes (Fig. 1). Esophagogastroduodenoscopy revealed no lesions in the stomach or duodenum. The patient was diagnosed as having jejunal cancer.

## 3. Treatment

### 3.1. First treatment

The patient was admitted to the Department of Surgery for an emergency exploratory laparotomy. Prior to surgery, a gynecological consultation was conducted to assess the necessity of diagnostic hysteroscopy. In September 2020, exploratory laparotomy was performed with the patient’s consent. Although the surgery initially started as a laparoscopic procedure, it had to be converted to laparotomy because of the presence of severe dense adhesions between the small bowel mesentery and a previous incision scar. Thorough examination of the entire small bowel was conducted, extending up to the ileocecal valve. Intraoperative exploration revealed a mass-like lesion in the proximal jejunum, which caused significant dilation of the proximal bowel, duodenum, and stomach.

**Figure 2.**
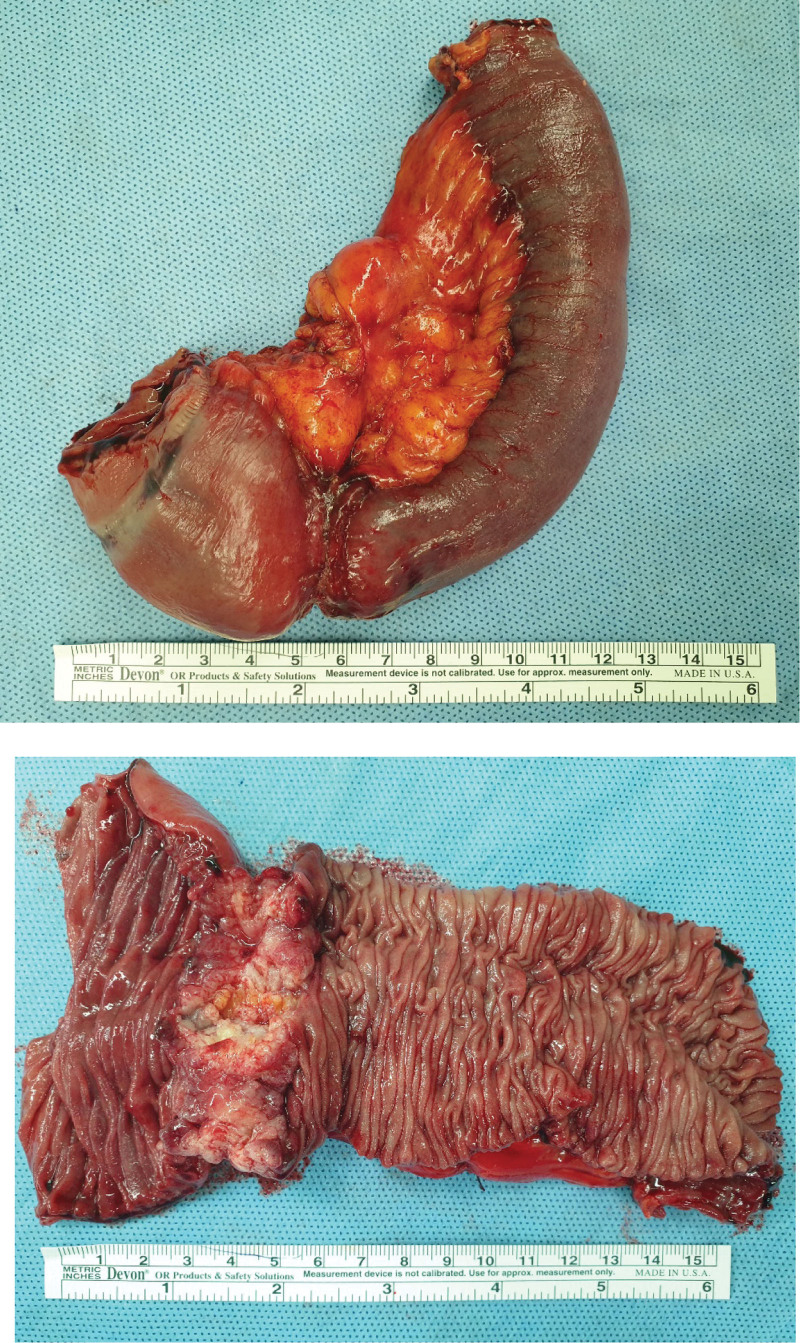
Gross specimen of small bowel resection.

To address this, a 15 cm segment of the proximal jejunum, including the lymph nodes at the root of the jejunal artery, was resected. Functional end-to-end anastomosis was performed to restore the intestinal continuity. The procedure was successfully concluded without any immediate complications, and postoperative management of the patient proceeded uneventfully. The surgical specimen obtained from jejunal resection contained a solid polypoid mass that completely obstructed the intestinal lumen (Fig. 2).

**Figure 3.**
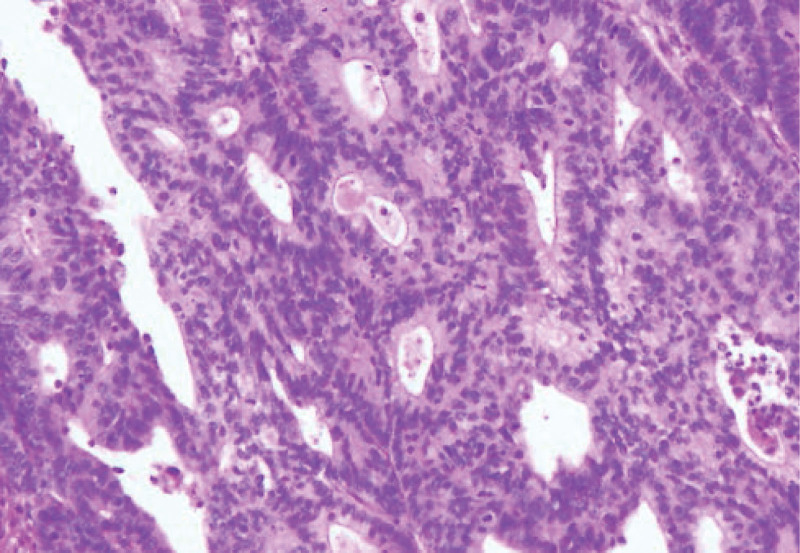
Microscopic findings of specimen.

**Figure 4.**
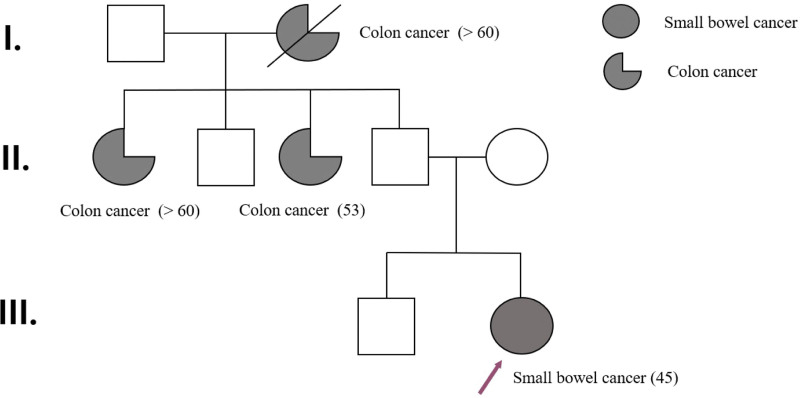
Genealogical tree of the family.

The histopathology report summary was T3N2M0 Stage IIIB SBC (adenocarcinoma, moderately differentiated (Fig. 3); metastasis of 4/17 lymph nodes) with lymphovascular invasion negative and perineural invasion present according to the Tumor-Node-Metastasis classification of malignant tumors.^[8]^ The surgical margins, including the proximal, distal, and mesenteric resection margins, were negative for tumor involvement. Following the initial diagnosis, further testing was performed to assess microsatellite instability (MSI) and to confirm the presence of deficient MMR. The results of the testing confirmed the presence of MSI-high, loss of expression of MLH1 and PMS2, and expression of MSH2 and MSH6. In addition, direct sequencing of the methylation status of CpG islands in the promoters of MLH1, MSH2, and MSH6 revealed a frameshift insertion specifically in MLH1 (c.1758dup, p.Met587Hisfs*6). Immunohistochemical analysis of programmed cell death ligand 1 (PD-L1) using the monoclonal antibody 22c3 was also conducted to identify a potential therapeutic option. The combined positive score was 20. Genetic profiling revealed that the tumor was epidermal growth factor receptor negative. Owing to the urgency of the initial operation, a thorough evaluation of the medical history of the patient’s relatives was conducted after the surgery (Fig. 4). Four family members, including the patient’s grandmother and 2 aunts, were affected by LS related cancers. All three of these family members, who were second-degree relatives of the patient, developed colon cancer when they were over the age of 50. LS was diagnosed based on the Amsterdam criteria II. Despite our offer of adjuvant chemotherapy, the patient declined the further treatment.

### 3.2. Second treatment

**Figure 5.**
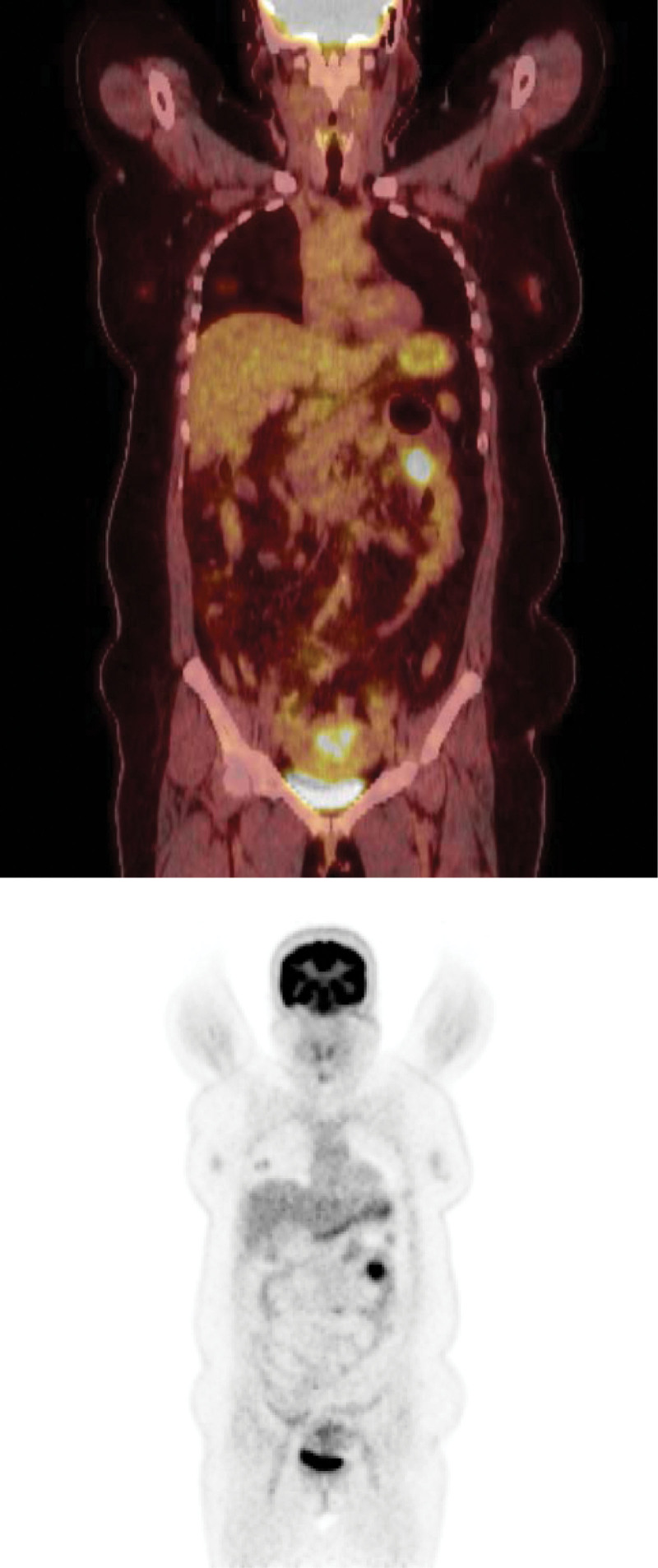
Coronal 18F-FDG PET CT Images. PET CT = Fluorine-18-fluorodeoxyglucose positron emission tomography.

During surveillance in November 2020, a metastatic mass was identified at the umbilicus using CT scan and Fluorine-18-fluorodeoxyglucose positron emission tomography (Fig. 5). Consequently, a secondary procedure involving mass excision, partial omentectomy, and adhesiolysis was performed. During laparotomy, a firm oval 3 cm × 2 cm mass and several enlarged nodes were observed in the jejunal mesentery near the previous anastomosis. En bloc resection was performed, involving removal of a 15cm segment of the mid-jejunum. Pathological analysis of the resected specimen revealed the presence of a moderately differentiated adenocarcinoma, with infiltration into the mesentery and metastasis detected in 4 out of the 19 resected lymph nodes. The resection margins were confirmed to be clear in all tumor cells.

In January 2021, chemotherapy with a combination of capecitabine-oxaliplatin was initiated. The patient declined pembrolizumab owing to the financial burden associated with its cost. The prescribed dosage for capecitabine was 1300 mg twice daily for a 2-week period, while a single dose of 240 mg oxaliplatin was administered once during each treatment cycle. After 8 cycles of capecitabine-oxaliplatin, subsequent cycles (9–12th) were carried out using a regimen that consisting of capecitabine alone. Chemotherapy was completed in September 2021; however, regular surveillance was continued. Chest and abdominal CT scans were performed every 3 months until March 2023. As of the submission date, the patient has remained stable with no signs of recurrence.

## 4. Discussion

SBCs are uncommon gastrointestinal malignancies that can occur sporadically or in association with certain predisposing conditions, including hereditary syndromes and immune-mediated intestinal disorders.^[9]^ It accounts for less than 5% of all gastrointestinal cancers, with the duodenum being the most commonly affected segment. Unlike CRC, research on the etiologic factors for SBC has been limited due to its rarity. However, it is interesting to note that the genetic predisposition to SBC has been relatively well studied, especially in specific hereditary syndromes such as FAP, LS, and Peutz–Jeghers syndrome.^[10,11]^

LS is associated with germline mutations in the DNA mismatch repair genes. It is widely recognized that hereditary mutations in MLH1, MSH2, MSH6, and PMS2 have been identified as causative factors in LS.^[11,12]^ The lifetime cancer risk for patients with LS is ≥80% with onset at a younger age and a tendency for more rapid progression.^[3]^ At the age of 80 years, individuals with LS have a cumulative risk of approximately 20% for developing CRC and 40% for developing endometrial cancer.^[3]^ Among patients with LS, the estimated risk of developing SBC is approximately 4%.^[12]^ In 2008, Koornstra et al^[12]^ reported that a significant proportion (34–78%), of patients with LS presented with SBC as their initial manifestation of the syndrome, and the diagnosis of LS was established using specific criteria known as the Amsterdam II or the revised Bethesda criteria. The opportunity to test for LS was limited to a specific group of patients who had been diagnosed with cancer. However, as tests for germline mutations have become popular, the National Institute of Health and Care Excellence has recommended “universal” testing for all individuals newly diagnosed with CRC and SBC since 2017.^[13,14]^ In this case, both MSI testing and a detailed pedigree-based evaluation confirmed the presence of LS.

Similar to CRC, the primary objective in the management of SBC in LS is to achieve complete resection (R0) of the localized tumor, including lymph node resection. Stage plays a crucial role as the most important prognostic factor in SBCs, similar to that in other types of cancer. Surgical resection is the primary treatment approach for SBCs. While the clinical management of SBCs is often based on the treatment outcomes and experience with CRC, SBC is increasingly recognized as a distinct category due to its lower incidence and generally poorer prognosis compared to CRC.

In cases of CRC diagnosed with LS, total or subtotal colectomy could be an option for surgical treatment. However, in cases of SBC, it is important to avoid extensive resection of the small bowel because of the potential risk of developing short bowel syndrome.

Despite the limited efficacy of chemotherapy in preventing cancer recurrence, it is crucial to make every effort to minimize the risk of recurrence. Previous studies have demonstrated that the administration of adjuvant therapy in patients who have already undergone R0 resection for SBC is associated with improved disease-free survival.^[15]^ In patients who are at a high risk of relapse, specifically defined as having a lymph node ratio (metastatic nodes/total harvested nodes) of 10% or higher, adjuvant therapy has been shown to improve overall survival but not disease-free survival.^[15]^ Recent studies have revealed the clinical efficacy of checkpoint inhibitors in the treatment of various solid tumors and hematologic malignancies. Specifically, inhibitors targeting programmed cell death protein 1, such as KEYTRUDA® (pembrolizumab), have demonstrated promising clinical benefits in several well-designed trials.^[2,16]^ In 2021, phase II trials were conducted to assess the effectiveness of pembrolizumab in patients with advanced small bowel cancer.^[2]^ However, the efficacy of pembrolizumab in treating SBCs is still under debated and controversial. To gain more clarity on the optimal chemotherapy regimen and potential benefits of Pembrolizumab in SBCs, further multicenter clinical trials are anticipated.^[17,18]^

The clinical symptoms of SBC with LS are typically asymptomatic or nonspecific. Furthermore, there are currently no widely accepted and effective population-based screening recommendations for SBC. As a result, SBCs are often diagnosed at more advanced stages, typically T3 and T4, when the symptoms become apparent. This advanced stage at diagnosis makes the treatment more challenging. Consequently, the prognosis for SBC is generally poor, with 5-year overall survival rates ranging from 14% to 33%. Moreover, there is a substantial difference in survival rates between different stages, with a more than 10-fold decrease in survival between stage I and stage IV cases.^[10,19]^ To the best of our knowledge, this is the first reported case of c.1758dup mutation in the MLH1 promoter in a patient with LS and SBC. This case emphasizes the importance of conducting additional investigations and offering genetic counseling to patients with LS who exhibit extracolonic manifestations. Additionally, routine screening for SBC in individuals with inherited genetic syndromes is generally recommended.^[12]^

## 5. Conclusion

SBC can serve as an initial manifestation of LS. SBC is a rare form of cancer, with a low incidence rate. However, when LS is suspected or diagnosed, additional tailored surveillance based on specific MMR gene mutations may be highly valuable.

## Acknowledgments

The authors would like to thank the patient and her family for providing informed consent for publication.

## Author contributions

**Conceptualization:** Donghyoun Lee.

**Data curation:** Donghyoun Lee, Jaemin Jo.

**Formal analysis:** Donghyoun Lee.

**Investigation:** Kyoung Won Yoon.

**Methodology:** Kyoung Won Yoon.

**Resources:** Kyoung Won Yoon.

**Software:** Kyoung Won Yoon.

**Supervision:** Jaemin Jo.

**Writing – original draft:** Kyoung Won Yoon.

**Writing – review & editing:** Donghyoun Lee, Jaemin Jo.
